# Antiacne and Anti-Inflammatory Effects of Phenolic Compounds from *Quercus acutissima* Carruth. Leaves

**DOI:** 10.1155/2022/9078475

**Published:** 2022-12-31

**Authors:** Eun Bin Kim, Eun Kyung Lee, Se Yeon Son, Min Won Lee

**Affiliations:** Laboratory of Pharmacognosy and Natural Product Derived Medicine, College of Pharmacy, Chung-Ang University, Seoul 06974, Republic of Korea

## Abstract

*Quercus* plants are widely distributed in Korea and have been used for their antiallergic and anti-inflammatory properties to treat dermatitis. The phenolic compounds of *Quercus acutissima* Carruth (QA) are estimated to have antioxidant and anti-inflammatory activities, based on the results of previous studies with *Quercus mongilica*, *Quercus stenophylla*, *Quercus gilva* Blame., and *Quercus acuta* Thunb. We yield QA extract and the isolated phenolic compounds (hyperoside (**1**), astragalin (**2**), kaempferol 3–O-(6″- galloyl)–*β*–D–glucopyranoside (KGG) (**3**), quercetin 3–O-(6″-O-galloyl)-*β*–D–glucopyranoside (QGG) (**4**), pedunculagin (**5**), and casuarinin (**6**)) and were identified using NMR. Among them, KGG (**3**) and QGG (**4**) were isolated for the first time from QA. QA extract and the isolated phenolic compounds demonstrated antioxidative, anti-inflammatory, and antiacne activities in RAW 264.7 mouse macrophage cells in vitro. **3**–**6** demonstrated strong inhibitory activities in the DPPH scavenging and NO production assay and anti-inflammatory and antiacne activities through western blotting (NLRP3, IL-1*β,* and 5*α*-reductase). The most outstanding activity in all experiments was casuarinin (**6**). The study findings suggest potential therapeutic candidates for acne.

## 1. Introduction

Natural products have contributed to mankind as an important source of treatment for various diseases and are used depending on traditional medicine or alternative medicine as a result of modern medicine's failure to accurately treat certain diseases. Today, with increased interest in natural-based drugs and functional foods, scientific knowledge such as exploring pharmacologically active components in natural products has increased. It has been found that several natural products have analgesic and anti-inflammatory effects [1–4].

*Quercus* is a genus of the Fagaceae family [5]with more than 600 species, many of which have medicinal properties [6, 7] that effectively treat eczema, furuncles, diarrhea, and tonsillitis [7]. The medicinal parts of the plant include the acorn [8, 9], roots [10], leaves [11, 12], stems [13], and bark [14]. *Quercus acutissima* Carruth. (QA) is a tall deciduous tree that grows up to 20 m in sunny, low mountainous regions and is widely dispersed in Korea. Its leaves are long and oval, with thorn-shaped serrations at the tip. The antiallergic, anti-inflammatory, and antiedema properties of QA have been used to treat dermatitis [15].

Acne vulgaris (AV) is a chronic inflammatory disease caused by excessive androgen-induced sebum production, which is thought to play a major role in the development of AV [16, 17]. Moreover, 5*α*-reductase activity in the skin influences the manifestation of excess circulating levels of endogenous androgen [17, 18], and sebum production is associated with the conversion of testosterone to dihydrotestosterone (DHT) through the action of 5*α*-reductase type 1 [19]. Inhibition of 5*α*-reductase type 1 (5*α*-R1) activity has been shown to decrease DHT levels and sebum production to help manage acne [20, 21]. The occurrence of acne, a chronic inflammatory disease, is likely to be reduced if the inflammatory response can be suppressed 5*α*-R1 activates DHT, which binds to the androgen receptor and causes acne. Inhibiting 5*α*-R1 expression may reverse or prevent acne.

The NLRP3 inflammasome pathway is reportedly activated by *P. acnes*, which has been recently associated with the pathogenesis of acne [22, 23]. *P. acnes* reportedly induced the secretion of a major inflammatory mediator, namely, interleukin‐1*β* (IL‐1*β*), from monocytes in a NACHT, LRR, and PYD domains‐containing protein 3 (NLRP3) - dependent manner [24, 25]. Inhibiting NLRP3 and IL-1*β* expression may also reverse or prevent acne. However, the role of *P. acnes* in the inflammatory response associated with AV remains unclear.

Previously we reported that phenolic compounds judged to be major components of the *Quercus* genus have antioxidant and anti-inflammatory activity [26, 27], and based on that, the goal of this study was to elucidate the structures of phenolic compounds isolated from QA leaves and further evaluate their antioxidant and anti-inflammatory activities including inhibitory activity against acne-associated proteins (NLRP3, IL-1*β*, and 5*α*-R1) in an effort to identify potential treatments for acne.

## 2. Materials and Methods

### 2.1. Plant Materials

QA leaves were collected from the Korea National Arboretum (Pocheon, South Korea) in June 2020, certificated by Ph. D Kim Sung-Sik, and a voucher specimen (QA 2020-01) was stored at the College of Pharmacy, Chung-Ang University, Korea.

### 2.2. General Experimental Procedure

Column chromatography was conducted using Amberlite® XAD®-2 resin column (20–50 *μ*m, Sigma-Aldrich, St. Louis, MO, USA), Sephadex LH-20 column (25–100 *μ*m, GE Healthcare Bio-Science AB, Uppsala, Sweden), ODS gel column (50 *μ*m, Daiso, Osaka, Japan), and MCI gel column (50 *μ*m, Mitsubishi Chemical, Tokyo, Japan).

Thin layer chromatography (TLC) of the compounds was performed on precoated silica gel 60 F_254_ plates (Merck, Darmstadt, Germany) in chloroform, methanol, and distilled water (6 : 4 : 1 volume ratio). Spots were detected under UV radiation (254 nm) and sprayed with FeCl_3_, 10% H_2_SO_4_, or anisaldehyde-H_2_SO_4_ followed by heating.

The chemical structures of the isolated compounds were determined using 1D-NMR, including ^1^H-(600 MHz) and ^13^C-(150 MHz) measurements, conducted using a JEOL NMR spectrometer (JEOL, Peabody, MA, USA) at Chung-Ang University.

### 2.3. Extraction and Isolation

QA leaves (1.7 kg) were extracted using 17 L 80% acetone at room temperature four times over three days. Acetone was removed under vacuum to yield 453.96 g of QA extract. The extract was dissolved in water and filtered through Celite 545 (Duksan Pure Chemical, Ansan, Korea). The filtrate (381.52 g) was then loaded onto an Amberlite® XAD®-2 resin column. Elution was performed with a gradient solvent system of H_2_O : MeOH (from 100 : 0, 50 : 50, to 0 : 100 volume ratio), yielding five fractions (from QA-1 to QA-5).

Fraction QA-4 (154.52 g) was separated using a Sephadex LH-20 column with a solvent gradient system of H_2_O : MeOH (from 100 : 0 to 0 : 100 volume ratio), yielding 8 subfractions (QA-4-1 to QA-4-8). The subfraction QA-4-8 (14.9438 g) was separated using an ODS gel column with a gradient solvent system of H_2_O : MeOH (100 : 0 to 0 : 100 volume ratio), yielding 11 subfractions (QA-4-8-1 to QA-4-8-11). Repeated column chromatography of subfraction QA-4-8-6 (1.1352 g) using an ODS gel column with a gradient solvent system of H_2_O : MeOH (100 : 0 to 0 : 100 volume ratio) yielded quercetin-3-O-galactoside (hyperoside, **1**, 362.3 mg).

The Subfraction QA-4-8-7 (2.7651 g) was applied to an MCI gel column, yielding kaempferol 3-O-*β*-D-glucopyranoside (astragalin, **2**, 1.39 g).

Subfraction QA-4-9 (9.5575 g) was separated using a Sephadex LH-20 column with a gradient solvent system of H_2_O : MeOH (100 : 0 to 0 : 100 volume ratio), yielding 6 subfractions (QA-4-9-1 to QA-4-9-6). Repeated column chromatography of subfraction QA-4-9-3 (2.8106 g) on an MCI gel column with a gradient solvent elution of H_2_O : MeOH (100 : 0 to 0 : 100 volume ratio) yielded kaempferol 3-O-(6″-galloyl)-*β*-D-glucopyranoside (KGG, **3**, 90.3 mg) and quercetin 3-O-(6″-O-galloyl)-*β*-D-glucopyranoside (QGG, **4**, 15.7 mg).

Subfraction QA-4-10 (26.0364 g) was applied to an ODS gel column with a gradient solvent system of H_2_O : MeOH (100 : 0 to 0 : 100 volume ratio) to yield pedunculagin (**5**, 3.4606 g).

Repeated column chromatography of subfraction QA-4-8-2 (1.2 g) using a Sephadex LH-20 column with a gradient solvent elution of H_2_O : MeOH (100 : 0 to 0 : 100 volume ratio) yielded casuarinin (**6**, 262.5 mg).

### 2.4. Measurement of DPPH Radical Scavenging Activity

The antioxidant activity of the isolated compounds was determined by evaluating each compound's scavenging activity of the stable 1,1-diphenyl-2-picrylhydazyl (DPPH) free radical (Sigma-Aldrich, St. Louis, MO, USA). Briefly, 20 *μ*L sample in anhydrous-ethanol was added to 180 *μ*L DPPH solution (0.2 mM in anhydrous-ethanol). After thoroughly mixing and reacting for 30 min, 37°C. The absorbance was measured at 490 nm using an enzyme-linked immunosorbent assay (ELISA) reader (Tecan Co. Ltd., Salzburg, Austria). L-Ascorbic acid was used as a positive control. The free radical scavenging activity was calculated as

(1) $$\begin{array}{c} Inhibition\;rate\;\left(\%\right)=100-\left(\frac{sample\;O.D.}{blank\;O.D.}\right)\times 100\text{.} \end{array}$$

IC_50_ values were defined as the concentration that could scavenge 50% DPPH free radical.

### 2.5. Cell Culture

Murine macrophage RAW 264.7 cells (Korea Cell Line Bank, Seoul, Korea) were cultivated at 37°C under a humidified atmosphere (approximately 5% CO_2_) in Dulbecco's modified Eagle's medium (DMEM; Sigma-Aldrich, St. Louis, MO, USA) containing 10% fetal bovine serum (FBS) (Welgene, Gyeongsangbuk-do, Korea), 100 IU/mL penicillin G, and 100 mg/mL streptomycin (Gibco BRL, Grand Island, NY, USA).

### 2.6. Measurement of Cell Viability

Cytotoxicity was measured via the mitochondrial-dependent reduction of 3-(4,5-dimethylthiazol-2-yl)-2,5-diphenyltetrazolium-bromide (MTT; Sigma-Aldrich, St. Louis, MO, USA) to purple formazan. RAW 264.7 cells (3 × 10^5^ cells/well) were seeded in a 96-well plate. After incubation at 37°C for 20 h, cells were reacted with 20 *μ*L of the sample. Serum-free DMEM was added 180 *μ*L to each well, and the plates were incubated at 37°C under a humidified atmosphere for 24 h. The medium was then gently removed and 100 *μ*L MTT solution (0.5 mg/mL) was added to each well. After incubation for 4 h, the supernatant was removed. The produced formazan was dissolved in 100 *μ*L dimethyl sulfoxide (DMSO), and the absorbance was measured at 540 nm using an ELISA reader (Tecan Co. Ltd., Salzburg, Australia). Relative cell viability was evaluated in accordance with the quantity of MTT converted to the insoluble formazan salt. As a control, distilled water was used instead sample. The optical density of the formazan generated in the control cells was considered to represent 100% viability. The results are presented as mean percentages of viable cells versus the respective control, calculated as

(2) $$\begin{array}{c} cell\;viability\;\left(\%\right)=\frac{sample\;O.D.}{control\;O.D.}\times 100\text{.} \end{array}$$

### 2.7. Measurement of Inhibitory Activity against NO Production

RAW 264.7 macrophage cells (3 × 10^5^ cells/well) were cultured in 96-well plates and incubated for 18 h at 37°C under a humidified atmosphere and approximately 5% CO_2_ and then treated with 20 *μ*L sample and 0.1 *μ*g/mL of lipopolysaccharide (LPS) (Sigma-Aldrich, St. Louis, MO, USA). After incubation for an additional 48 h, the NO concentration was evaluated using the Griess assay. After getting the supernatant, Griess reagent (0.1% naphthyl ethylenediamine and 1% sulfanilamide in 5% H_3_PO_4_ solution; Sigma-Aldrich, St. Louis, MO, USA) was added to each well. The NO content was determined by measuring the absorbance at 540 nm against a standard sodium nitrite curve. NO production inhibitory activity was calculated as equation (3). IC50 values were defined as the concentration that could inhibit 50% of NO production.

(3) $$\begin{array}{c} Inhibition\;rate\;\left(\%\right)=\left[1-\frac{\left(sample\;O.D.–blank\;O.D.\right)}{\left(control\;O.D.–blank\;O.D.\right)}\right]\times 100\text{.} \end{array}$$

### 2.8. Western Blotting

RAW 264.7 cells (2 × 10^6^ cells/well) were preincubated in 6-well plates for 18 h and then treated with a sample and 1 *μ*g/mL LPS. After incubation for 46 h, the cells were harvested and washed thrice with phosphate-buffered saline (PBS). Cell lysates were prepared using RIPA buffer (50 mM/L Tris–HCl (pH 7.4), 150 mM/L NaCl, 1% Triton X-100, 0.1% sodium dodecyl sulfate (SDS), and 1 mM/L ethylenediaminetetraacetic acid) (Thermo Fisher Scientific, Waltham, MA, USA) for 30 min on ice. The cell lysates were centrifuged at 15,814 ×g for 15 min at 4°C, and then 50 *μ*g cell lysate was subjected to electrophoresis using SDS-polyacrylamide gels (10–12%). The extracted proteins were transferred onto a polyvinylidene fluoride (PVDF) membrane (Bio-Rad, Hercules, CA, USA), which was blocked with a blocking buffer (Thermo Fisher Scientific) for 30–60 min at room temperature. Subsequently, the membrane was incubated with IL-1*β* (1 : 1000; Cell Signaling Technology, MA, USA), NLRP3 antibodies (1 : 1000; Cell Signaling Technology, MA, USA) and 5*α*-R1(1 : 200; Santa Cruz, CA, USA) overnight at 4°C with gentle shaking. After washing the membrane three times with tris-buffered saline containing 0.1% Tween-20, the membrane was incubated with horseradish peroxidase-linked antirabbit IgG secondary antibody (1 : 3000; Cell Signaling Technology, MA, USA) and m-IgG*κ* binding protein (BP)-HRP (1 : 1000; Santa Cruz, CA, USA) for 1 h at room temperature. Bands were visualized using the LAS- 500 luminescent image analyzer (GE Healthcare Life Sciences, NJ, USA) using ECL solution (GE Healthcare, Chicago, Illinois, USA). Band intensity was analyzed using the ImageJ software (National Institutes of Health, Bethesda, MD, USA).

### 2.9. Statistical Analyses

All data were analyzed using one-way analysis of variance (ANOVA) followed by the Student–Newman–Keuls test with the SPSS software pack (IBM, Armonk, NY, USA). Differences between groups were considered to be significantly different when the *p* value was less than 0.05. Values bearing different superscripts in the same column are significantly distinct from other data.

## 3. Results and Discussion

### 3.1. Phytochemicals Isolated from QA

**Figure 1:**
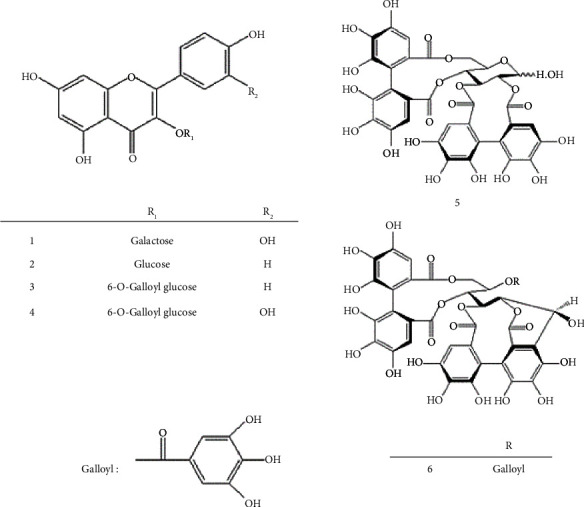
Compounds **1**–**6** isolated from QA leaves.

The 80% acetone extract of QA leaves yielded four flavonoids (hyperoside (**1**), astragalin (**2**), kaempferol 3–O-(6″- galloyl)–*β*–D–glucopyranoside (KGG) (**3**), and quercetin 3–O-(6″-O-galloyl)-*β*–D–glucopyranoside (QGG) (**4**)) and two ellagitannins (pedunculagin (**5**) and casuarinin (**6**)). Two of which (**3** and **4**) were isolated from QA for the first time (Figure 1).

#### 3.1.1. Compound **1**

**1** was obtained as a yellow powder. TLC analysis revealed a spot under UV radiation, a dark blue spot was detected after FeCl_3_ spraying, and a yellow spot was detected after 10% H_2_SO_4_ spraying followed by heating.

The 1H-NMR spectrum of **1** exhibited an ABX-spin system with a meta-coupled aromatic signal at *δ* 7.89 (1H, d, *J* = 1.2 Hz, H-2′), an ortho-coupled aromatic signal at *δ* 6.91 (1H, d, *J* = 8.4 Hz, H-5′) and an ortho-meta-coupled aromatic signal at *δ* 7.54 (1H, dd, *J* = 1.8, 8.4 Hz, H-6′). Two aromatic protons were indicated by meta-coupled signals at *δ* 6.22 (1H, d, *J* = 2 Hz, H-6) and 6.47 (1H, d, *J* = 2 Hz, H-8). These results confirmed the identity of **1** as quercetin [28].

Furthermore, the ^1^H and ^13^C-NMR spectra of **1** suggested the presence of a galactosyl moiety with signals at *δ* 5.36 (1H, d, *J* = 7.8 Hz, Gal-1), 3.25–3.92 (5H, m, Gal-2, 3, 4, 5 and 6) and 102.33 (Gal-1), 74.61 (Gal-2), 76.99 (Gal-3), 70.43 (Gal-4), 78.04 (Gal-5), and 61.47 (Gal-6). The large coupling constant of the anomeric proton at *δ* 5.25 (*J* = 7.2 Hz, gal-1) indicated the *β*-configuration for galactose. Therefore, compound **1** was identified as quercetin-3-O-*β*–D-galactose (hyperoside) by comparing the spectral data with values reported in the literature [29].

#### 3.1.2. Compound **2**

**2** was obtained as a yellow powder. TLC analysis revealed a spot under UV radiation and a dark blue spot was detected after FeCl_3_ spraying. Dark brown spots were also detected after 10% H_2_SO_4_ spraying and anisaldehyde-H_2_SO_4_ spraying followed by heating.

The ^1^H-NMR spectrum of **2** exhibited an aromatic A_2_B_2_-spin system at *δ*H 8.11 (2H, d, *J* = 8.8, H-2′,6′) and 6.94 (2H, d, *J* = 8.8, H-3′, 5′) of B-ring with aromatic signals at *δ*H 6.47 (1H, s, H-8) and 6.24 (1H, s, H-6) of A-ring. These results confirmed the identity of **2** as kaempferol. The proton signals at *δ*H 3.28–4.38 (4H, m, H-2″, -3″, -4″, -5″) confirmed the presence of a glucose moiety in **2**, which was supported by ^13^C-NMR signals at *δ*C 103.99 (C-1″), 74.61 (C-2″), 77.05 (C-3″), 70.39 (C-4″), 77.20 (C-5″), and 61.95 (C-6″) [30]. Comparing the data with values reported in the literature, **2** was identified as kaempferol 3-O-*β*-D-glucopyranoside (astragalin) [31].

#### 3.1.3. Compound **3**

**3** was obtained as a yellow amorphous powder. During TLC analysis, a green spot was detected after FeCl_3_ spraying, whereas a yellow spot was detected after 10% H_2_SO_4_ spraying followed by heating.

In the ^1^H-NMR spectrum, **3** was exhibited a similar to that of **2** aromatic A_2_B_2_-type system with aromatic signals at *δ*H 8.05 (2H, d, *J* = 9.0 Hz, H-2′, 6′) and 6.782 (2H, d, *J* = 9.0 Hz, H-3′, 5′) of B-ring and meta-coupled signals at *δ*H 6.46 (1H, d, *J* = 1.9 Hz, H-8 and 6.23 (1H, d, *J* = 1.9 Hz, H-6) of A-ring with glucosyl moiety (at *δ*H 5.30 (1H, d, *J* = 7.8 Hz, Glc H-1), 4.30 (1H, s, Glc H-6″a), 3.58 (1H, dd, *J* = 7.8, 7.8 Hz, Glc H-6″b), and 3.29–3.49 (Glc H-2″, -3″, -4″, and -5″)) which was supported by ^13^C-NMR signals at *δ*C 103.98 (Glc C-1), 77.13 (Glc C-3), 74.64 (Glc C-2, C-5), 69.85 (Glc C-4), and 63.06 (Glc C-6) [26]. The ^13^C-NMR spectrum of **3** also demonstrated a galloyl moiety at *δ*C 160.17 (C-4′), 131.26 (C-2′, 6′), 121.43 (C-1′), and 115.12 (C-3′, 5′). These data implied that the B-ring was a phenol moiety with a hydroxyl group at C-4′. Finally, a galloyl group with a large singlet signal at *δ*H 7.04 (H-2‴ and H-6‴) was identified as adjacent to the 6-position of glucose due to the downfield 6″-proton signal at *δ*H 4.30. Taken together, **3** was identified as kaempferol-3-O-*β*-D-(6″-O-galloyl)-glucopyranoside (KGG) [32].

#### 3.1.4. Compound **4**

**4** was obtained as a yellow amorphous powder. TLC analysis revealed a light-yellow spot after 10% H_2_SO_4_ spraying followed by heating.

The ^1^H-NMR spectrum of **4** was very similar to that of **1**, demonstrating typical flavonoid signals. A meta-coupled doublet signal at *δ*H 7.69 (1H, d, *J* = 2.4 Hz, H-2′), an ortho-coupled doublet signal at *δ*H 6.71 (1H, d, *J* = 8.4 Hz, H-5′), and an ortho-meta-coupled signal at *δ*H 7.68 (1H, dd, *J* = 10.2, 2.8 Hz, H-6′) suggested a 3′,4′-dihydroxylated pattern for a B-ring in the ABX system. Two meta-coupled doublet signals were observed at *δ*H 6.24 (1H, d, *J* = 2.4 Hz, H-6)] and 6.46 (1H, d, *J* = 2.4 Hz, H-8), referring to a 5,7-dihydroxylated pattern for an A-ring. These data confirmed the identity of the aglycone as quercetin. An anomeric proton signal was also detected at *δ*H 5.33 (1H, d, *J* = 7.8 Hz, H-1″). The relatively large coupling constant of the anomeric proton signal indicated a *β*-D-configuration of the glycoside bond. Proton signals at *δ*H 3.47–4.32 (H-2″, -3″, -4″, -5″, -6″*α*, and -6″*β*) suggested the six nonanomeric protons of glucose. The ^13^C-NMR spectrum of **4** also suggested the existence of a glucosyl moiety with signals at *δ*C 103.76 (C-1″), 77.23 (C-3″), 74.80 (C-5″) 74.61 (C-2″), 69.90 (C-4″), and 63.17 (C-6″)). Finally, a galloyl group with a large singlet signal at *δ*H 7.04 (2H, s, H-2‴, and H-6‴) was determined to be adjacent to the 6-position of the sugar due to the downfield-shifted 6″ protons at *δ*H 4.33 and 4.31 [26].

From the above results, **4** was characterized as quercetin-3-O-*β*- D-(6″-O-galloyl)-glucopyranoside and in comparison with the informed spectral data in the literature [26, 33].

#### 3.1.5. Compound **5**

**5** was obtained as a brown amorphous powder. TLC analysis revealed a brown spot after 10% H_2_SO_4_ spraying followed by heating.

In NMR analysis, all signals for **5** were duplicated, and H-1 proton signals were observed below 5.43 ppm, suggesting that **5** existed as a mixture of two isomers caused by an unacylated anomeric center on the sugar molecule. The ^1^H-NMR spectrum of **5** exhibited signals corresponding to two hexahydroxydiphenoyl (HHDP) groups at *δ*H 6.30, 6.31, 6.48, 6.53, 6.57, 6.58, 6.64, 6.65 (8H in total, s, HHDP -2, 2″) in the aromatic region, with a ^4^C_1_ conformation of the glucose core corresponding to signals at *δ*H 3.74–5.46 and large coupling constants in the sugar region. The chemical shifts at *δ*H 5.03 (H-4), 5.21–5.26 (H-6a″), and separated signals at *δ*H 3.75–3.82 (H-6b″) of glucosyl moiety suggested that the HHDP group should be connected at 4 and 6 of the glucose core [26]. Furthermore, the upfield-shifted H-1 of the *β*-anomer, corresponding to a signal at *δ*H 5.05 (1/2H, d, *J* = 7.8 Hz, H-1″), compared to that of the *α*-anomer, corresponding to a signal at *δ*H 5.45 (1/2H, d, *J* = 3.6 Hz, H-1″), indicated an anisotropic effect in H-1 [26]. Based on these results, **5** was identified as pedunculagin [34].

#### 3.1.6. Compound **6**

**6** was obtained as a brown amorphous powder. TLC analysis revealed a brown spot after 10% H_2_SO_4_ spraying followed by heating.

The ^1^H-NMR spectrum of **6** showed similarity with **5** except for the presence of one galloyl group moiety at *δ* 7.13 (2H, S, galloyl–H), and the ^13^C-NMR spectrum of **6** also demonstrated a galloyl moiety at *δ*C 139.53 (C-4), 108.53 (C-2, 6), 121.75 (C-1), and 146.38 (C-3, 5). Glucose group signals were observed at *δ* 5.52 (1H, dd, *J* = 2.0, 8.4 Hz, Glc-4″), 5.36 (1H, d, *J* = 4.8 Hz, Glc-1″), 5.11 (1H, dd, *J* = 2.0, 5.1 Hz, Glc-2″), 5.09 (1H, t, *J* = 2.0 Hz, Glc-3″), 4.87 (1H, dd, *J* = 3.0, 8.4 Hz, Glc-5″), 4.71 (1H, dd, *J* = 3.0, 12.0 Hz, H-6″b), and 3.96 (1H, d, *J* = 12.0 Hz, H-6″a). In the ^1^H-NMR spectrum of **6**, especially, H1 anomeric-proton was detected at 5.36 (1H, d, *J* = 4.8 Hz) as *β*-configuration indicating the glucose core is opened [35]. And, **6** also indicated the presence of a glucose moiety with signals at *δ*C 65.46 (Glc-1″), 72.80 (Glc-2″), 66.56 (Glc-3″), 70.52 (Glc-4″), 68.19 (Glc-5″), and 64.59(Glc-6″) in the ^13^C-NMR spectrum. From these results, **6** was identified as casuarinin by comparing the spectral data with values reported in the literature [35, 36].

### 3.2. DPPH Radical Scavenging Activity

The antioxidant activities of the QA extract and the six isolated compounds (**1**–**6**) were assessed by measuring their 1,1-diphenyl-2-picrylhydazyl (DPPH) free radical scavenging activity.

**Figure 2:**
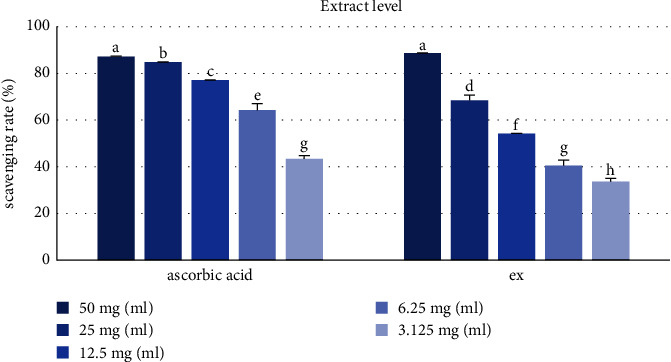
DPPH scavenging rate of the QA extract. L-ascorbic acid was used in the control. Ex, extract. Different letters denote significant differences (*p* < 0.05) between concentrations and groups.

**Figure 3:**
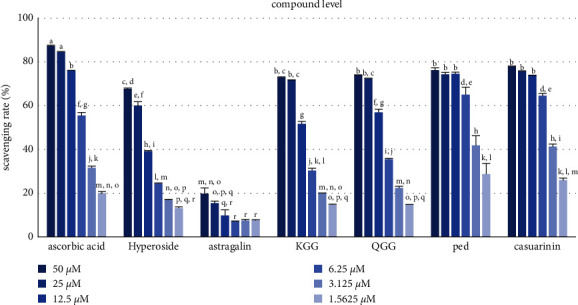
DPPH scavenging rate of compounds (1–6) isolated from QA. L-ascorbic acid was used in the control. Hyperoside (**1**); astragalin (**2**); KGG (**3**); QGG (**4**); ped, pedunculagin (**5**); casuarinin (**6**). Different letters denote significant differences (*p* < 0.05) between concentrations and groups.

DPPH is easily supplied with hydrogen because hydrazine nitrogen is unstable, and antioxidant activity can be measured through the loss of its purple color when abundant hydrogen is supplied from antioxidants [37] that provide hydrogen to reduce the stable radical DPPH to yellow nonradical diphenyl-picrylhydrazine (DPPH-H) [38]. The IC_50_ value indicates that the lower the value, the higher the antioxidant activity. The QA extract (IC_50_ = 6.70 ± 0.50 *μ*g/mL) possessed potent DPPH radical scavenging activity compared with L-ascorbic acid (IC_50_ = 5.25 ± 1.67 *μ*g/m) (Table 1, Figure 2). The isolated compounds (**1**–**6**) also indicated potent DPPH radical scavenging activity. In particular, **5** (IC_50_ = 9.96 ± 0.39 *μ*M) and **6** (IC_50_ = 10.41 ± 0.27 *μ*M) showed more effective free radical scavenging activities than L-ascorbic acid (IC_50_ = 11.69 ± 0.21 *μ*M) used positive control (Table 2, Figure 3).

### 3.3. Cell Viability

**Figure 4:**
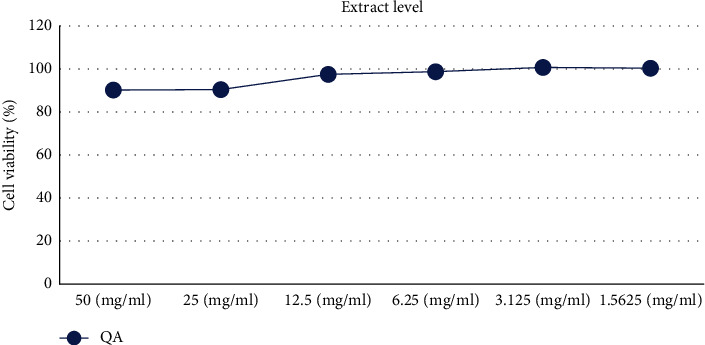
Effects of QA extract on RAW 264.7 cell viability.

**Figure 5:**
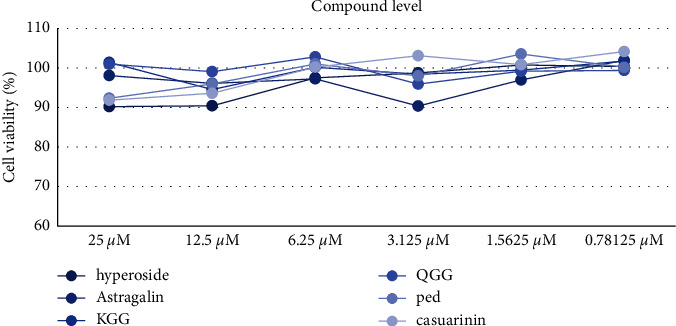
Effects of compounds isolated from QA on RAW 264.7 cell viability. Hyperoside (**1**), astragalin (**2**), KGG (**3)**, QGG (**4**), ped, pedunculagin (**5**), and casuarinin (**6**).

MTT assays, based on the mitochondria-dependent reduction of lightly colored tetrazolium salt to formazan of the intense violet color [39, 40], were performed to assess the cytotoxic and cell viability effects of QA extract and its compounds. The QA extract was performed at experimental doses (50, 25, 12.5, 6.25, 3.125, and 1.5625 *μ*g/mL) and compounds were performed at experimental doses (25, 12.5, 6.25, 3.125, and 1.5625 *μ*M) in RAW 264.7 cells. The cell viability did not affect (>80%) at experimental doses of QA extract and compounds. These results suggest that the inhibitory activities against NO production and the expression of acne-associated cytokines and proteins were not associated with their cytotoxic effects (Figures 4 and 5).

### 3.4. Inhibitory Activity against NO Production

**Figure 6:**
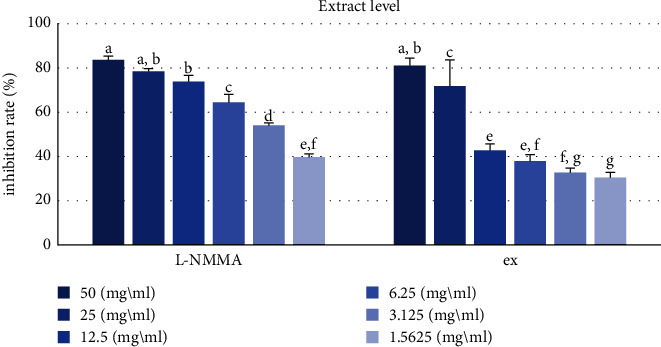
Effects of QA extract on inhibition of NO production. L-NMMA was used as a positive control. Ex, extract. Different letters denote significant differences (*p* < 0.05) between concentrations and groups.

**Figure 7:**
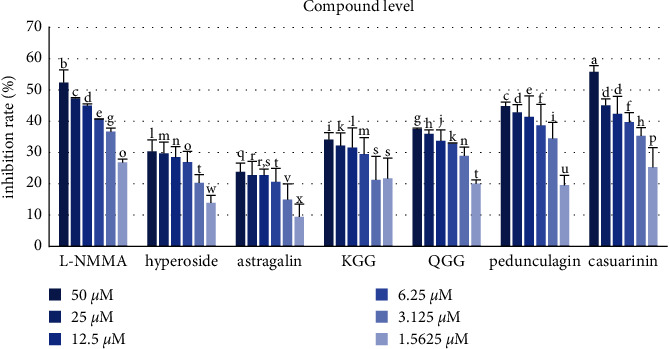
Inhibitory effects of isolated compounds against NO production. L-NMMA was used as a positive control. Hyperoside (**1**), astragalin (**2**), KGG (**3)**, QGG (**4**), pedunculagin (**5**), and casuarinin (**6**). Different letters denote significant differences (*p* < 0.05) between concentrations and groups.

The role of nitro oxide (NO), a free radical produced in mammalian cells, has been reported to have been extensively studied. Overproduction of NO is associated with acute or chronic inflammation [41, 42], so inhibition of NO production can be a useful treatment strategy [43]. Inhibitory activity against NO production was evaluated in RAW 246.7 macrophage cells. QA extract (IC_50_ = 16.84 ± 1.42 *μ*g mL) exerted an inhibitory effect against NO production (Table 3, Figure 6). Among the six compounds isolated from QA, **6** (IC_50_ = 34.56 ± 1.36 *μ*M) exhibited the strongest inhibitory activity against NO production, which was significantly stronger than that of the positive control, namely, L-NMMA (IC_50_ = 38.32 ± 0.23 *μ*M) (Table 4, Figure 7).

### 3.5. Inhibitory Activity against Acne-Associated Proteins (NLRP3, IL-1*β*, and 5*α*-R1)

Through DPPH radical scavenging and NO production inhibitory activities guided isolation from QA, we isolated six compounds (**1**–**6**). Then, we selected compounds (**3**–**6**) that showed potent activity compared with other compounds for the evaluation of antiacne.

In relation to acne, NLRP3 is activated by *P.acnes*, which releases activated IL-1*β*, resulting in localized inflammation as neutrophils become enriched [44]. Testosterone also turns into dihydrotestosterone, causing acne. The inhibitory effects of compounds (**3**–**6**) against acne-associated proteins (NLRP3, IL-1*β*, 5*α*-R1) were evaluated by western blotting.

**Figure 8:**
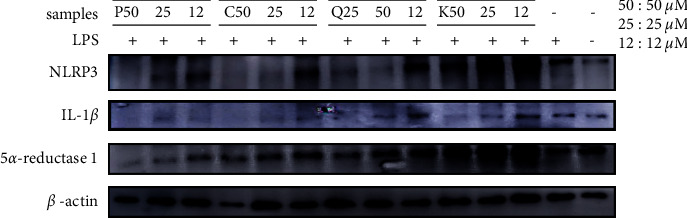
Effect of K (KGG, 3), Q (QGG, 4), P (pedunculagin, 5), and C (casuarinin, 6) isolated from QA on LPS-induced production of NLRP3, IL-1*β*, and 5*α*-R1 protein in RAW 264.7 cells. The results are expressed as mean ± SD of triplicate tests. ^*∗*^*p* < 0.05, ^*∗∗*^*p* < 0.01.

The results demonstrated that compounds (**3**–**6**) inhibited the expression of NLRP3 protein in RAW 264.7 cells. Moreover, treatment with 50 *μ*M QGG (**4**) and casuarinin (**6**) achieved the highest NLRP3 protein inhibitory activities. Treatment with the isolated compounds (**3**–**6**) significantly inhibited IL-1*β* protein expression in RAW 264.7 cells in a concentration-dependent manner. Furthermore, pedunculagin (**5**) possessed the most potent 5*α*-R1 protein inhibitory activity compared to the other compounds and the control (Figure 8).

## 4. Discussion

We aimed to evaluate the inhibition of the antioxidant, anti-inflammatory, and acne-associated factors of the extracts of QA and isolated compounds from QA. Acne is one of the chronic diseases and is caused by various causes. Excessive sebum production causes acne, and sebum production is related to 5*α*-reductase. 5*α*-reductase changes DHT from testosterone and it has isoenzymes which are 5*α*-reductase type 1 and type 2 [45]. The presence of 5*α*-reductase type 1 (5*α*-R1) in the skin may imply that the androgen regulation of sebaceous glands and sebum production requires the local conversion of testosterone to dihydrotestosterone (DHT) [46]. Skin 5*α*-reductase activity is a primary factor influencing the manifestation of endogenous androgen excess [18]. Sebum production is associated with DHT which changes from testosterone by 5*α*-reductase type 1 [19]. Inhibition of 5*α*-R1 was found to help manage acne by reducing DHT levels and sebum production. Also, Activation of the NLRP3 inflammasome is closely associated with various chronic diseases [47]. The role of inflammasome activation by *Propionibacterium acnes* in the pathogenesis of acne was only recently proposed [48]. Since NLRP3 is the first mediator in the activation of the inflammasome pathway, inhibition of NLRP3 expression may inhibit the next step of the pathway, in which caspase-1 finally activates IL-1*β* [24], resulting in the development of acne. Inhibiting NLRP3 and IL-1*β* expression may reverse or prevent acne.

The *Quercus* genus reported several types of bioactivities such as antioxidant, anti-inflammatory, antimicrobial, and antibacterial. Therefore, we have confirmed from previous studies that use as a treatment for inflammatory diseases by evaluating inhibition of NO production and proinflammatory cytokines from *Quercus gilva* Blume [49] and inhibition of 5a-reductase and anti-inflammatory activity from *Quercus mongolica* [50].

The results of our previous studies suggested that QA has the potential to be used as a treatment for inflammatory diseases such as acne.

Our results in this current study demonstrated that the QA as well as compounds **1**–**6** exhibited good antioxidant ability. Especially, ellagitannins (**5**, **6**) were the most potent. In addition, the QA and compounds exerted anti-inflammatory effects. Based on these results, we selected compounds and evaluated whether compounds were able to inhibit the expression of NLRP3, IL-1*β*, 5*α* -R1 associated acne. Several studies have shown that phenolic compounds inhibit the activation of NLRP3 inflammasome and inhibit the expression of various inflammatory factors such as IL-1*β* [51, 52]. In addition, our previous studies confirmed that phenolic compounds effectively inhibit 5*α*-reductase [50, 53]. Pedunculagin (**5**) has the effect of the most 5*α*-R1 expression inhibition and casuarinin (**6**), and QGG (**4**) was demonstrated the most inhibitory effects in NLRP3 expression through western blot analysis, IL-1*β* was suppressed to a normal level in all compounds. The compound that showed the most excellent activity in all biology activities was casuarinin (**6**). Casuarinin (**6**) has a structure in which one more galloyl group is attached to the pedunculagin (**5**), compounds (**5** and **6**) have previously been found in *Alnus sieboldiana* and *Casuarina stricta* [35, 54], so it is demonstrated to have better activity than pedunculagin (**5**) due to its structural stability.

In this study, we demonstrated the effects of the biological activity of *Quercus acutissima* Carruth. Leaves extracts and isolated compounds by evaluating the inhibition of expression of inflammatory mediated proteins associated with acne, DPPH radical scavenging activity, and inhibition of NO production. Our results suggested that *Quercus acutissima* Carruth. Leave extracts and isolated compounds from QA have therapeutic functions and values as new treatment materials for anti-inflammatory healthy functional foods, acne treatments, and cosmetics for acne skin.

## 5. Conclusions

The QA leaf extract yielded six phenolic compounds (hyperoside (**1**), astragalin (**2**), KGG (**3**), QGG (**4**), pedunculagin (**5**), and casuarinin (**6**)). DPPH radical scavenging activity and inhibitory activity against NO production were evaluated in vitro. **3**–**6** exhibited superior DPPH radical scavenging activity and inhibitory activity against NO production. Selected compounds (**3**–**6**) were evaluated for the inhibitory effect on acne-associated protein expression. **3**–**6** inhibited IL-1*β* protein expression on a dose-dependent RAW 264.7 cells, and **4** and **6** possessed the highest NLRP3 protein inhibitory activity. **5** exhibited the highest inhibitory activity against 5*α*-R1 protein expression. According to the results, several isolated compounds possessed antioxidative, anti-inflammatory, and antiacne activities, providing potential candidates for the development of acne treatment agents.

## Figures and Tables

**Table 1:** IC_50_ values of DPPH radical scavenging activity for QA extract.

Test sample	IC_50_ (*μ*g/mL)
QA	6.70 ± 0.50^b^
L-ascorbic acid	5.25 ± 1.67^a^

**Table 2:** IC_50_ values of DPPH radical scavenging activity for isolated compounds from QA.

Compound	IC_50_ (*μ*M)
Hyperoside (**1**)	26.32 ± 0.59^c^
Astragalin (**2**)	>100^d^
Kaempferol 3–O-(6″- galloyl)–*β*–D–glucopyranoside (**3,** KGG)	20.49 ± 0.24^b^
Quercetin 3–O-(6″-O-galloyl)-*β*–D–glucopyranoside (**4**, QGG)	18.87 ± 0.29^b^
Pedunculagin (**5**)	9.96 ± 0.39^a^
Casuarinin (**6**)	10.41 ± 0.27^a^
L-ascorbic acid	11.69 ± 0.21^a^

**Table 3:** IC_50_ values of inhibitory activity against NO production for QA extract.

IC_50_ (mg/mL)	
L-NMMA	2.46 ± 0.77
QA	16.84 ± 1.42

**Table 4:** IC_50_ values of inhibitory activity against NO production for isolated compounds.

IC_50_ (*μ*M)	
L-NMMA	38.32 ± 0.23^b^
**1**	82.17 ± 1.23^f^
**2**	93.30 ± 2.64^g^
**3**	76.35 ± 2.29^e^
**4**	55.33 ± 0.30^d^
**5**	48.14 ± 1.12^c^
**6**	34.56 ± 1.36^a^

## Data Availability

The data used to support the findings of this study are included within the article.

## Supplementary Materials

Supplementary Materials Supplementary Materials are provided files including the NMR spectra of six compounds isolated from QA. Figure S1-1: ^1^H-NMR spectrum of compound **1** (600 MHz, Acetone-d_6_+D_2_O); Figure S1-2: ^13^C-NMR spectrum of compound **1** (150 MHz, DMSO- d_6_+D_2_O); Figure S2-1: ^1^H-NMR spectrum of compound **2** (600 MHz, Acetone-d_6_); Figure S2-2: ^13^C-NMR spectrum of compound **2** (150 MHz, Acetone-d_6_); Figure S3-1: ^1^H-NMR spectrum of compound **3** (600 MHz, Acetone-d_6_); Figure S3-2: ^13^C-NMR spectrum of compound **3** (150 MHz, Acetone-d_6_); Figure S4-1: ^1^H-NMR spectrum of compound **4** (600 MHz, Acetone-d_6_); Figure S4-2: ^13^C-NMR spectrum of compound **4** (150 MHz, Acetone-d_6_); Figure S5-1: ^1^H-NMR spectrum of compound **5** (600 MHz, Acetone-d_6_); Figure S5-2: ^13^C-NMR spectrum of compound **5** (150 MHz, Acetone-d_6_); Figure S6-1: ^1^H-NMR spectrum of compound **6** (600 MHz, Acetone-d_6_); and Figure S6-2: ^13^C-NMR spectrum of compound **6** (150 MHz, Acetone-d_6_).
