# Supplementary figures and images for: Antiacne and Anti-Inflammatory Effects of Phenolic Compounds from Quercus acutissima Carruth. Leaves
Source: Evid Based Complement Alternat Med. 2022 Dec 31;2022:9078475. doi: 10.1155/2022/9078475 (PMC9825228; doi:10.1155/2022/9078475)

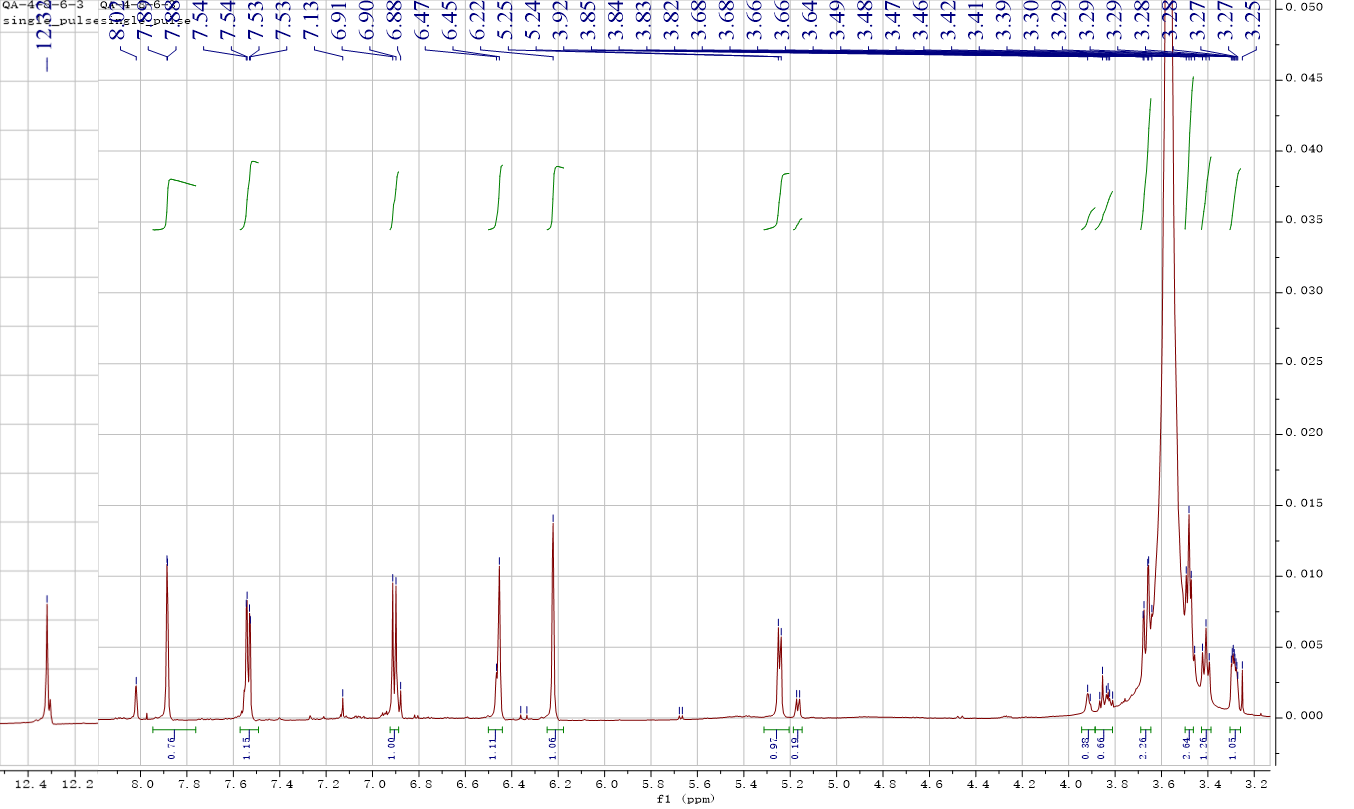

Supplement: Supplementary Materials — are provided files including the NMR spectra of six compounds isolated from QA. Figure S1-1: 1H-NMR spectrum of compound 1 (600 MHz, Acetone-d6+D2O); Figure S1-2: 13C-NMR spectrum of compound 1 (150 MHz, DMSO- d6+D2O); Figure S2-1: 1H-NMR spectrum of compound 2 (600 MHz, Acetone-d6); Figure S2-2: 13C-NMR spectrum of compound 2 (150 MHz, Acetone-d6); Figure S3-1: 1H-NMR spectrum of compound 3 (600 MHz, Acetone-d6); Figure S3-2: 13C-NMR spectrum of compound 3 (150 MHz, Acetone-d6); Figure S4-1: 1H-NMR spectrum of compound 4 (600 MHz, Acetone-d6); Figure S4-2: 13C-NMR spectrum of compound 4 (150 MHz, Acetone-d6); Figure S5-1: 1H-NMR spectrum of compound 5 (600 MHz, Acetone-d6); Figure S5-2: 13C-NMR spectrum of compound 5 (150 MHz, Acetone-d6); Figure S6-1: 1H-NMR spectrum of compound 6 (600 MHz, Acetone-d6); and Figure S6-2: 13C-NMR spectrum of compound 6 (150 MHz, Acetone-d6). [file 9078475.f1.zip › Figure S1-1.png]

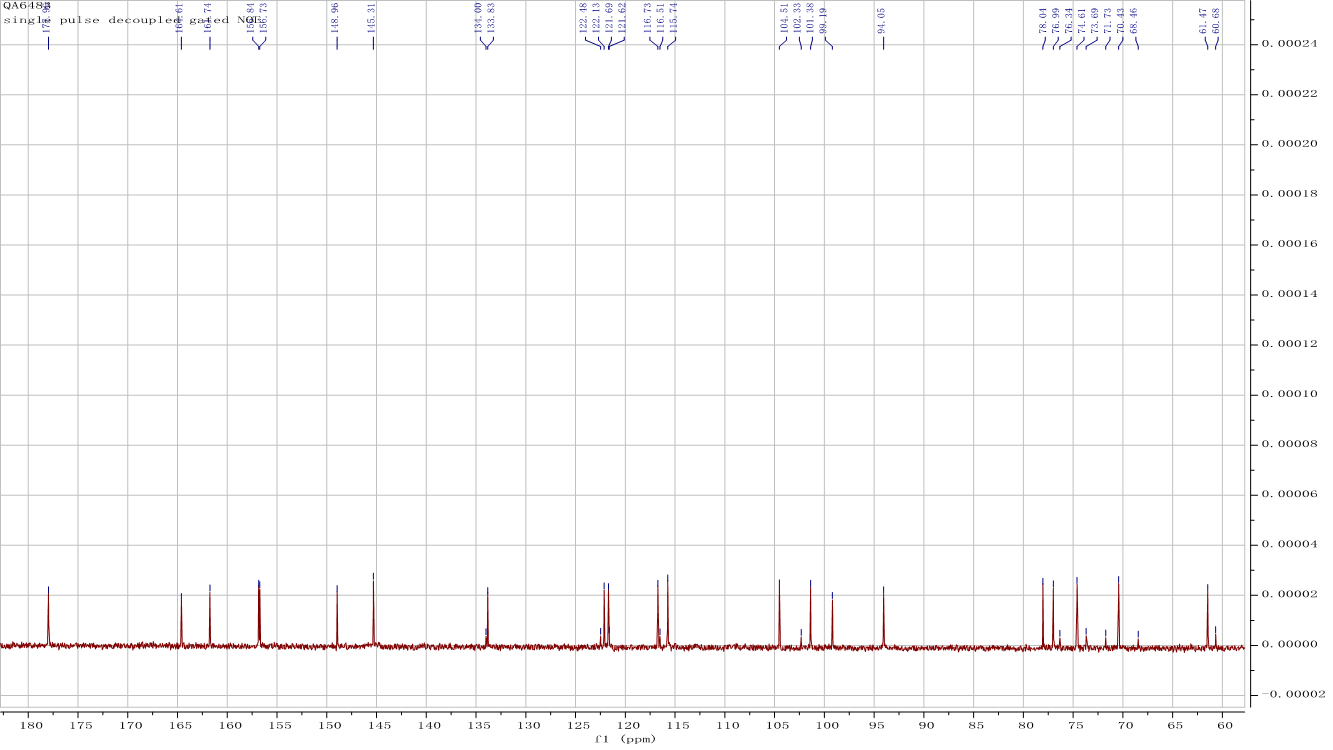

Supplement: Supplementary Materials — are provided files including the NMR spectra of six compounds isolated from QA. Figure S1-1: 1H-NMR spectrum of compound 1 (600 MHz, Acetone-d6+D2O); Figure S1-2: 13C-NMR spectrum of compound 1 (150 MHz, DMSO- d6+D2O); Figure S2-1: 1H-NMR spectrum of compound 2 (600 MHz, Acetone-d6); Figure S2-2: 13C-NMR spectrum of compound 2 (150 MHz, Acetone-d6); Figure S3-1: 1H-NMR spectrum of compound 3 (600 MHz, Acetone-d6); Figure S3-2: 13C-NMR spectrum of compound 3 (150 MHz, Acetone-d6); Figure S4-1: 1H-NMR spectrum of compound 4 (600 MHz, Acetone-d6); Figure S4-2: 13C-NMR spectrum of compound 4 (150 MHz, Acetone-d6); Figure S5-1: 1H-NMR spectrum of compound 5 (600 MHz, Acetone-d6); Figure S5-2: 13C-NMR spectrum of compound 5 (150 MHz, Acetone-d6); Figure S6-1: 1H-NMR spectrum of compound 6 (600 MHz, Acetone-d6); and Figure S6-2: 13C-NMR spectrum of compound 6 (150 MHz, Acetone-d6). [file 9078475.f1.zip › Figure S1-2.png]

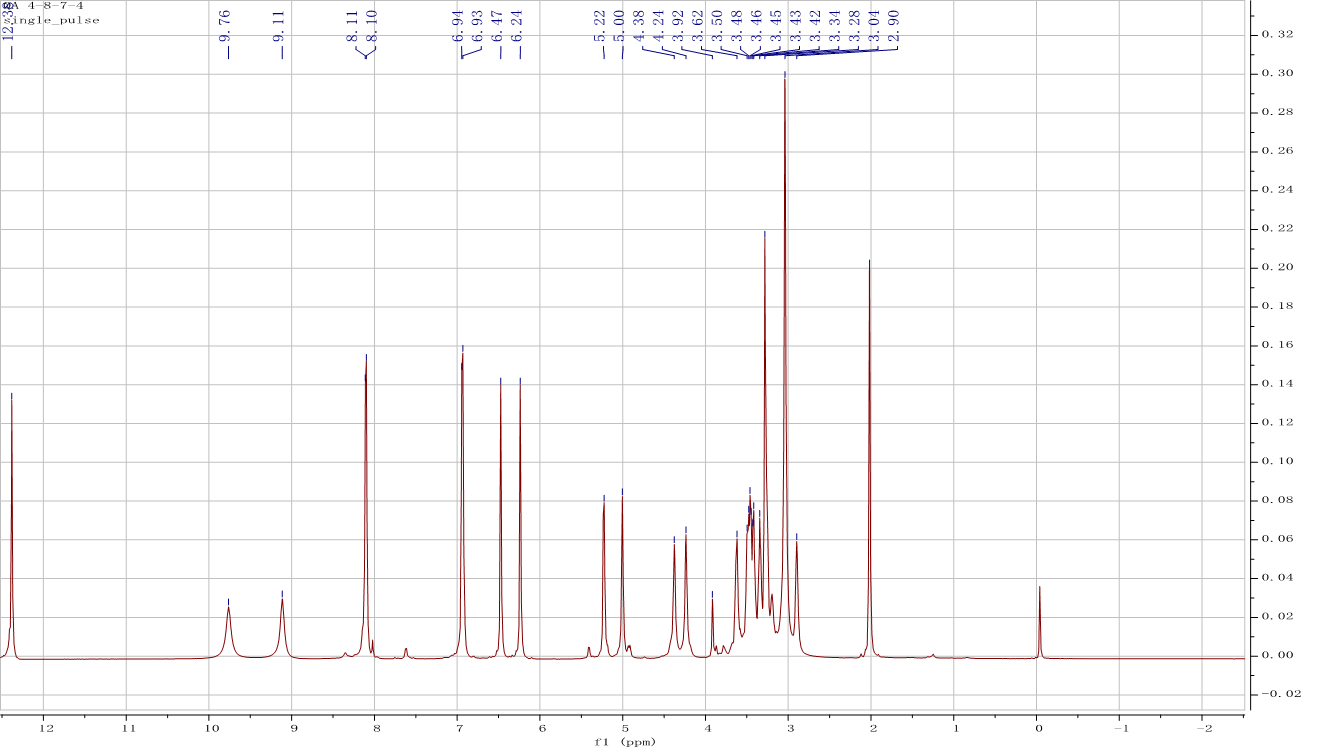

Supplement: Supplementary Materials — are provided files including the NMR spectra of six compounds isolated from QA. Figure S1-1: 1H-NMR spectrum of compound 1 (600 MHz, Acetone-d6+D2O); Figure S1-2: 13C-NMR spectrum of compound 1 (150 MHz, DMSO- d6+D2O); Figure S2-1: 1H-NMR spectrum of compound 2 (600 MHz, Acetone-d6); Figure S2-2: 13C-NMR spectrum of compound 2 (150 MHz, Acetone-d6); Figure S3-1: 1H-NMR spectrum of compound 3 (600 MHz, Acetone-d6); Figure S3-2: 13C-NMR spectrum of compound 3 (150 MHz, Acetone-d6); Figure S4-1: 1H-NMR spectrum of compound 4 (600 MHz, Acetone-d6); Figure S4-2: 13C-NMR spectrum of compound 4 (150 MHz, Acetone-d6); Figure S5-1: 1H-NMR spectrum of compound 5 (600 MHz, Acetone-d6); Figure S5-2: 13C-NMR spectrum of compound 5 (150 MHz, Acetone-d6); Figure S6-1: 1H-NMR spectrum of compound 6 (600 MHz, Acetone-d6); and Figure S6-2: 13C-NMR spectrum of compound 6 (150 MHz, Acetone-d6). [file 9078475.f1.zip › Figure S2-1.png]

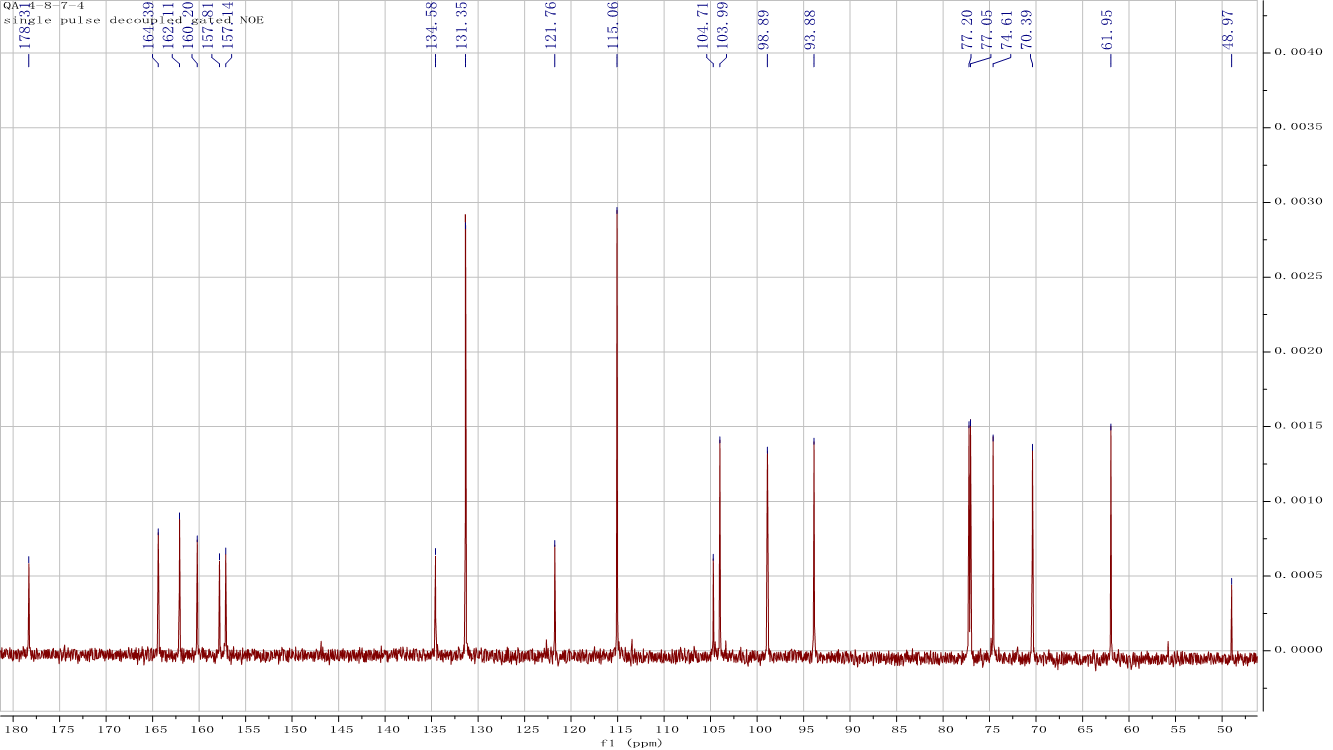

Supplement: Supplementary Materials — are provided files including the NMR spectra of six compounds isolated from QA. Figure S1-1: 1H-NMR spectrum of compound 1 (600 MHz, Acetone-d6+D2O); Figure S1-2: 13C-NMR spectrum of compound 1 (150 MHz, DMSO- d6+D2O); Figure S2-1: 1H-NMR spectrum of compound 2 (600 MHz, Acetone-d6); Figure S2-2: 13C-NMR spectrum of compound 2 (150 MHz, Acetone-d6); Figure S3-1: 1H-NMR spectrum of compound 3 (600 MHz, Acetone-d6); Figure S3-2: 13C-NMR spectrum of compound 3 (150 MHz, Acetone-d6); Figure S4-1: 1H-NMR spectrum of compound 4 (600 MHz, Acetone-d6); Figure S4-2: 13C-NMR spectrum of compound 4 (150 MHz, Acetone-d6); Figure S5-1: 1H-NMR spectrum of compound 5 (600 MHz, Acetone-d6); Figure S5-2: 13C-NMR spectrum of compound 5 (150 MHz, Acetone-d6); Figure S6-1: 1H-NMR spectrum of compound 6 (600 MHz, Acetone-d6); and Figure S6-2: 13C-NMR spectrum of compound 6 (150 MHz, Acetone-d6). [file 9078475.f1.zip › Figure S2-2.png]

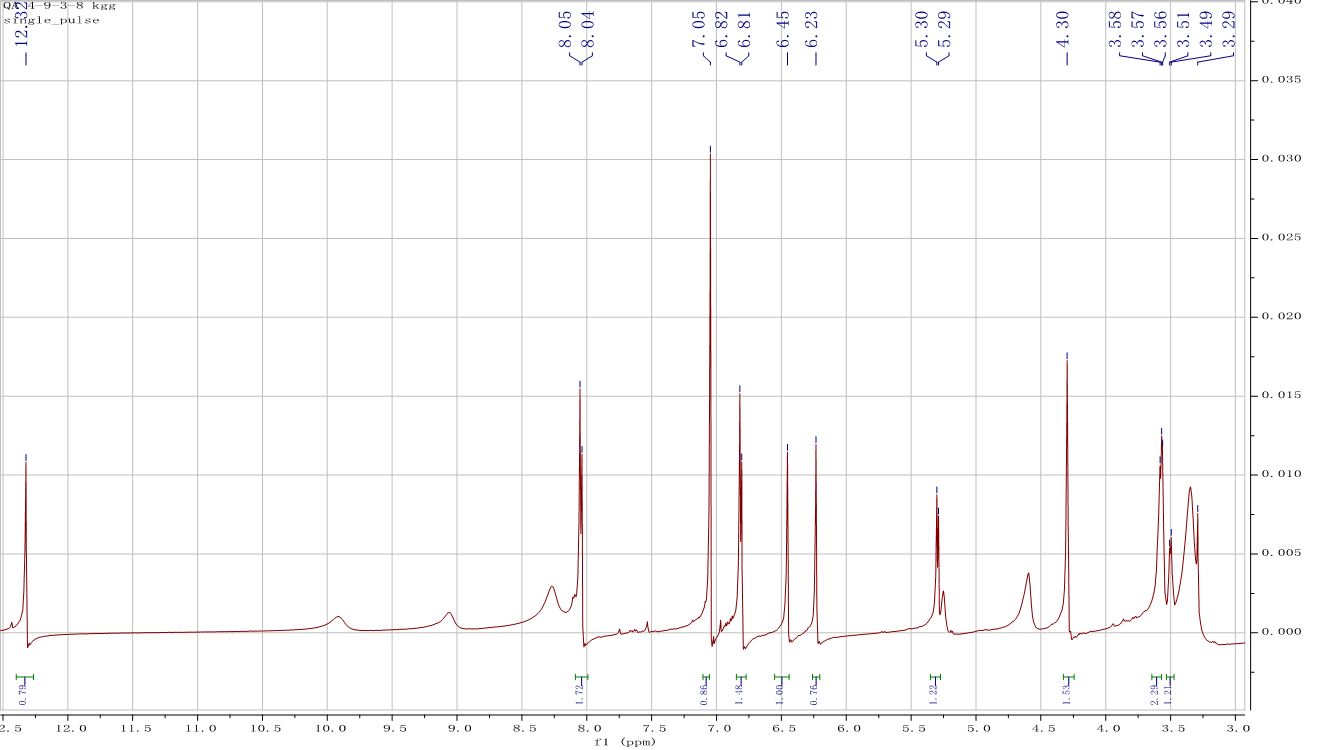

Supplement: Supplementary Materials — are provided files including the NMR spectra of six compounds isolated from QA. Figure S1-1: 1H-NMR spectrum of compound 1 (600 MHz, Acetone-d6+D2O); Figure S1-2: 13C-NMR spectrum of compound 1 (150 MHz, DMSO- d6+D2O); Figure S2-1: 1H-NMR spectrum of compound 2 (600 MHz, Acetone-d6); Figure S2-2: 13C-NMR spectrum of compound 2 (150 MHz, Acetone-d6); Figure S3-1: 1H-NMR spectrum of compound 3 (600 MHz, Acetone-d6); Figure S3-2: 13C-NMR spectrum of compound 3 (150 MHz, Acetone-d6); Figure S4-1: 1H-NMR spectrum of compound 4 (600 MHz, Acetone-d6); Figure S4-2: 13C-NMR spectrum of compound 4 (150 MHz, Acetone-d6); Figure S5-1: 1H-NMR spectrum of compound 5 (600 MHz, Acetone-d6); Figure S5-2: 13C-NMR spectrum of compound 5 (150 MHz, Acetone-d6); Figure S6-1: 1H-NMR spectrum of compound 6 (600 MHz, Acetone-d6); and Figure S6-2: 13C-NMR spectrum of compound 6 (150 MHz, Acetone-d6). [file 9078475.f1.zip › Figure S3-1.png]

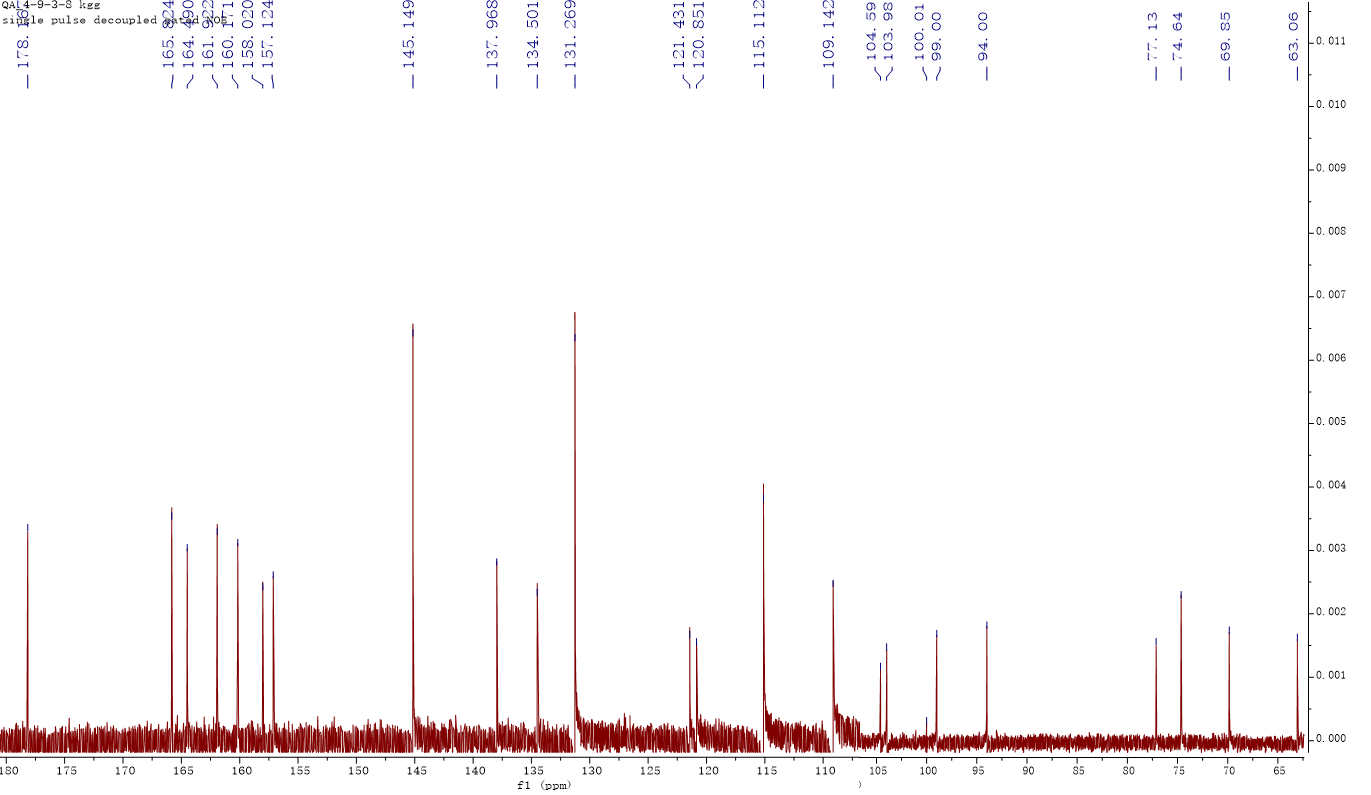

Supplement: Supplementary Materials — are provided files including the NMR spectra of six compounds isolated from QA. Figure S1-1: 1H-NMR spectrum of compound 1 (600 MHz, Acetone-d6+D2O); Figure S1-2: 13C-NMR spectrum of compound 1 (150 MHz, DMSO- d6+D2O); Figure S2-1: 1H-NMR spectrum of compound 2 (600 MHz, Acetone-d6); Figure S2-2: 13C-NMR spectrum of compound 2 (150 MHz, Acetone-d6); Figure S3-1: 1H-NMR spectrum of compound 3 (600 MHz, Acetone-d6); Figure S3-2: 13C-NMR spectrum of compound 3 (150 MHz, Acetone-d6); Figure S4-1: 1H-NMR spectrum of compound 4 (600 MHz, Acetone-d6); Figure S4-2: 13C-NMR spectrum of compound 4 (150 MHz, Acetone-d6); Figure S5-1: 1H-NMR spectrum of compound 5 (600 MHz, Acetone-d6); Figure S5-2: 13C-NMR spectrum of compound 5 (150 MHz, Acetone-d6); Figure S6-1: 1H-NMR spectrum of compound 6 (600 MHz, Acetone-d6); and Figure S6-2: 13C-NMR spectrum of compound 6 (150 MHz, Acetone-d6). [file 9078475.f1.zip › Figure S3-2.png]

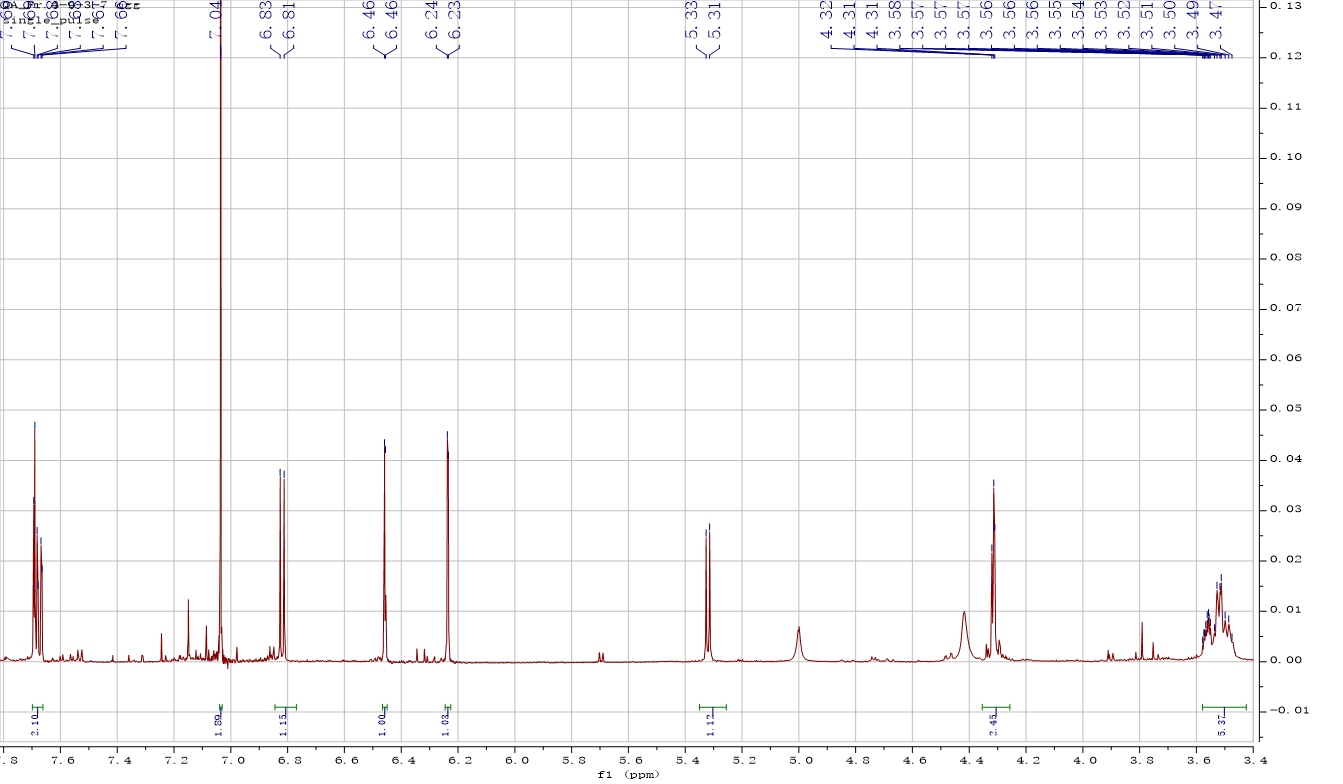

Supplement: Supplementary Materials — are provided files including the NMR spectra of six compounds isolated from QA. Figure S1-1: 1H-NMR spectrum of compound 1 (600 MHz, Acetone-d6+D2O); Figure S1-2: 13C-NMR spectrum of compound 1 (150 MHz, DMSO- d6+D2O); Figure S2-1: 1H-NMR spectrum of compound 2 (600 MHz, Acetone-d6); Figure S2-2: 13C-NMR spectrum of compound 2 (150 MHz, Acetone-d6); Figure S3-1: 1H-NMR spectrum of compound 3 (600 MHz, Acetone-d6); Figure S3-2: 13C-NMR spectrum of compound 3 (150 MHz, Acetone-d6); Figure S4-1: 1H-NMR spectrum of compound 4 (600 MHz, Acetone-d6); Figure S4-2: 13C-NMR spectrum of compound 4 (150 MHz, Acetone-d6); Figure S5-1: 1H-NMR spectrum of compound 5 (600 MHz, Acetone-d6); Figure S5-2: 13C-NMR spectrum of compound 5 (150 MHz, Acetone-d6); Figure S6-1: 1H-NMR spectrum of compound 6 (600 MHz, Acetone-d6); and Figure S6-2: 13C-NMR spectrum of compound 6 (150 MHz, Acetone-d6). [file 9078475.f1.zip › Figure S4-1.png]

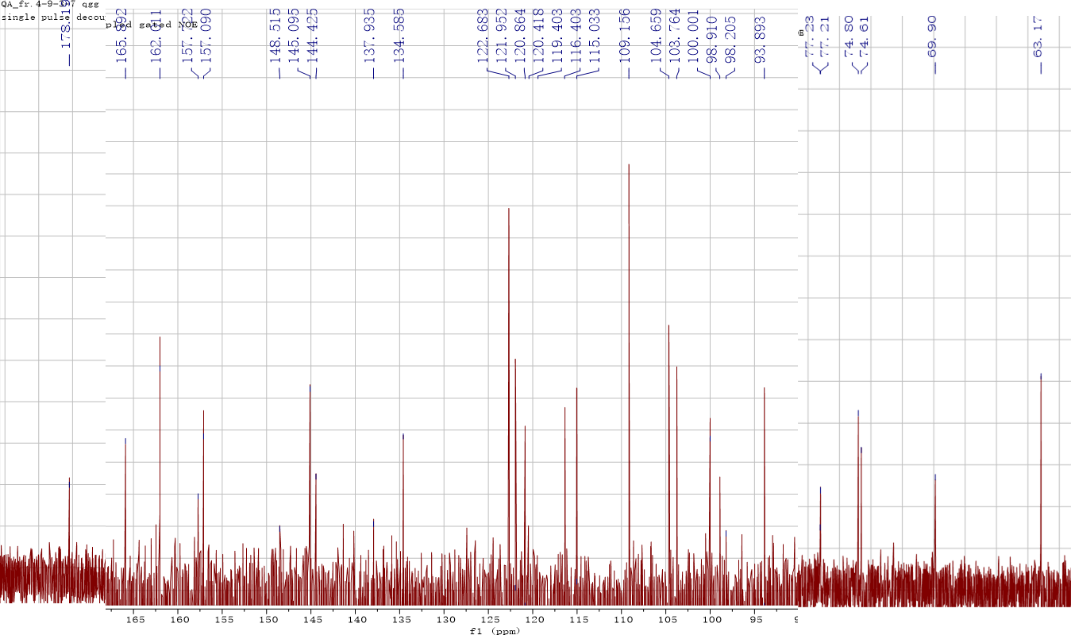

Supplement: Supplementary Materials — are provided files including the NMR spectra of six compounds isolated from QA. Figure S1-1: 1H-NMR spectrum of compound 1 (600 MHz, Acetone-d6+D2O); Figure S1-2: 13C-NMR spectrum of compound 1 (150 MHz, DMSO- d6+D2O); Figure S2-1: 1H-NMR spectrum of compound 2 (600 MHz, Acetone-d6); Figure S2-2: 13C-NMR spectrum of compound 2 (150 MHz, Acetone-d6); Figure S3-1: 1H-NMR spectrum of compound 3 (600 MHz, Acetone-d6); Figure S3-2: 13C-NMR spectrum of compound 3 (150 MHz, Acetone-d6); Figure S4-1: 1H-NMR spectrum of compound 4 (600 MHz, Acetone-d6); Figure S4-2: 13C-NMR spectrum of compound 4 (150 MHz, Acetone-d6); Figure S5-1: 1H-NMR spectrum of compound 5 (600 MHz, Acetone-d6); Figure S5-2: 13C-NMR spectrum of compound 5 (150 MHz, Acetone-d6); Figure S6-1: 1H-NMR spectrum of compound 6 (600 MHz, Acetone-d6); and Figure S6-2: 13C-NMR spectrum of compound 6 (150 MHz, Acetone-d6). [file 9078475.f1.zip › Figure S4-2.png]

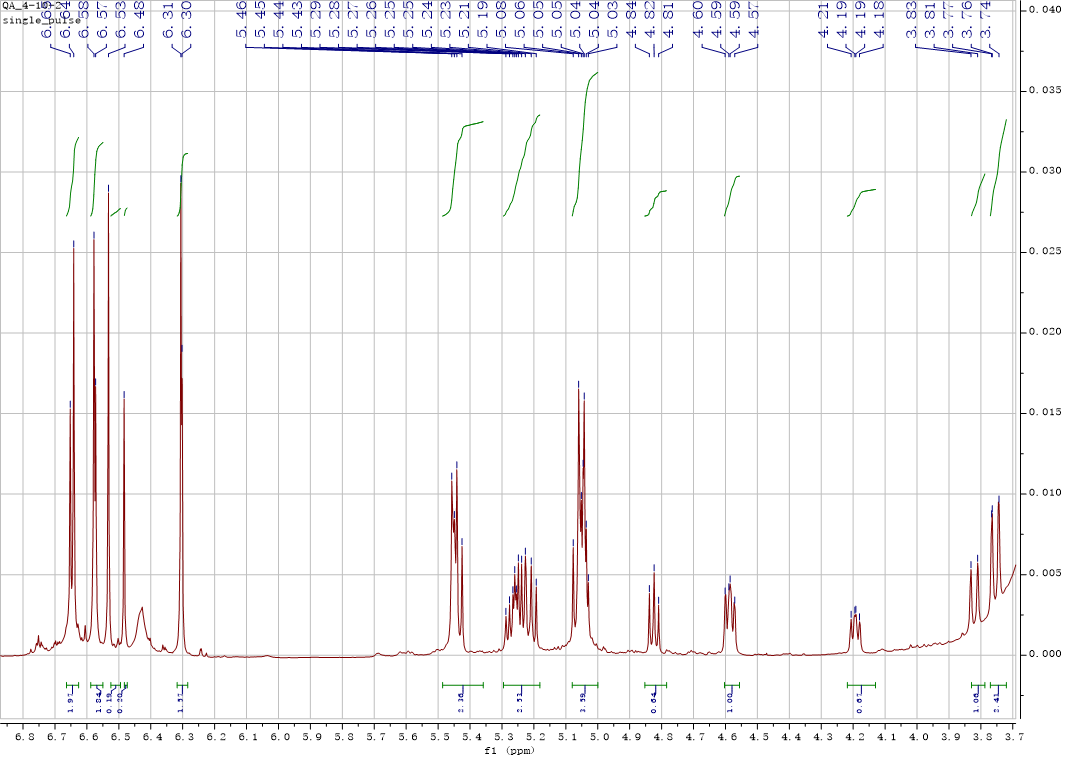

Supplement: Supplementary Materials — are provided files including the NMR spectra of six compounds isolated from QA. Figure S1-1: 1H-NMR spectrum of compound 1 (600 MHz, Acetone-d6+D2O); Figure S1-2: 13C-NMR spectrum of compound 1 (150 MHz, DMSO- d6+D2O); Figure S2-1: 1H-NMR spectrum of compound 2 (600 MHz, Acetone-d6); Figure S2-2: 13C-NMR spectrum of compound 2 (150 MHz, Acetone-d6); Figure S3-1: 1H-NMR spectrum of compound 3 (600 MHz, Acetone-d6); Figure S3-2: 13C-NMR spectrum of compound 3 (150 MHz, Acetone-d6); Figure S4-1: 1H-NMR spectrum of compound 4 (600 MHz, Acetone-d6); Figure S4-2: 13C-NMR spectrum of compound 4 (150 MHz, Acetone-d6); Figure S5-1: 1H-NMR spectrum of compound 5 (600 MHz, Acetone-d6); Figure S5-2: 13C-NMR spectrum of compound 5 (150 MHz, Acetone-d6); Figure S6-1: 1H-NMR spectrum of compound 6 (600 MHz, Acetone-d6); and Figure S6-2: 13C-NMR spectrum of compound 6 (150 MHz, Acetone-d6). [file 9078475.f1.zip › Figure S5-1.png]

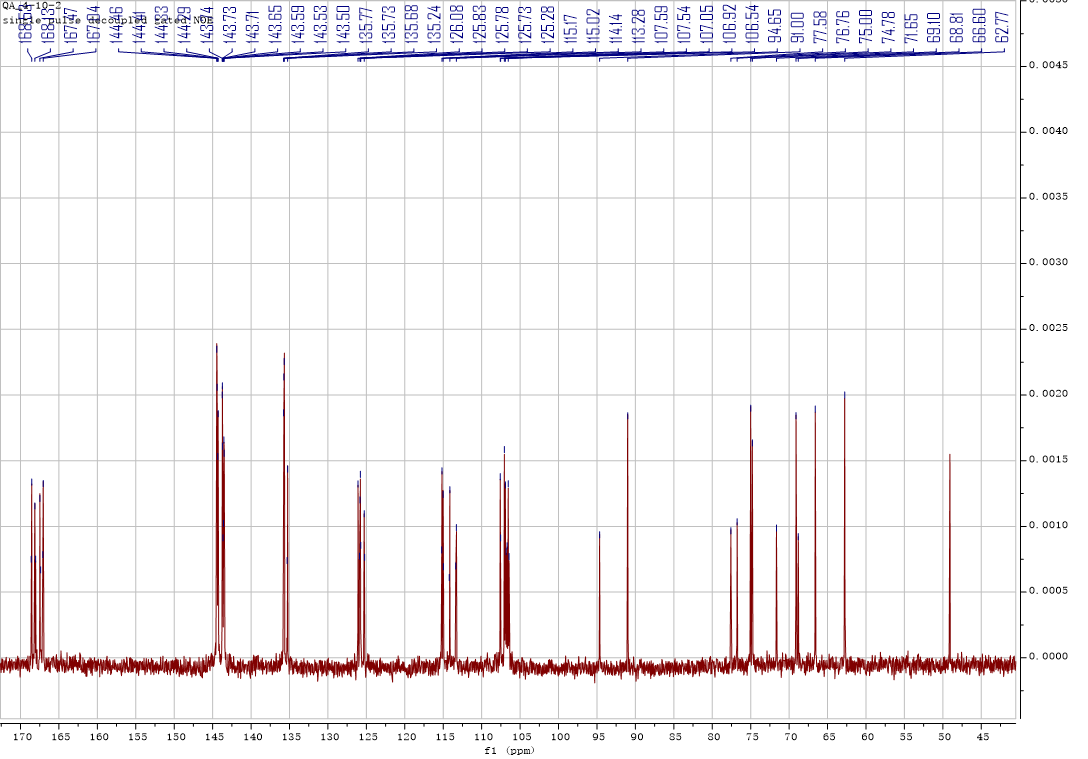

Supplement: Supplementary Materials — are provided files including the NMR spectra of six compounds isolated from QA. Figure S1-1: 1H-NMR spectrum of compound 1 (600 MHz, Acetone-d6+D2O); Figure S1-2: 13C-NMR spectrum of compound 1 (150 MHz, DMSO- d6+D2O); Figure S2-1: 1H-NMR spectrum of compound 2 (600 MHz, Acetone-d6); Figure S2-2: 13C-NMR spectrum of compound 2 (150 MHz, Acetone-d6); Figure S3-1: 1H-NMR spectrum of compound 3 (600 MHz, Acetone-d6); Figure S3-2: 13C-NMR spectrum of compound 3 (150 MHz, Acetone-d6); Figure S4-1: 1H-NMR spectrum of compound 4 (600 MHz, Acetone-d6); Figure S4-2: 13C-NMR spectrum of compound 4 (150 MHz, Acetone-d6); Figure S5-1: 1H-NMR spectrum of compound 5 (600 MHz, Acetone-d6); Figure S5-2: 13C-NMR spectrum of compound 5 (150 MHz, Acetone-d6); Figure S6-1: 1H-NMR spectrum of compound 6 (600 MHz, Acetone-d6); and Figure S6-2: 13C-NMR spectrum of compound 6 (150 MHz, Acetone-d6). [file 9078475.f1.zip › Figure S5-2.png]

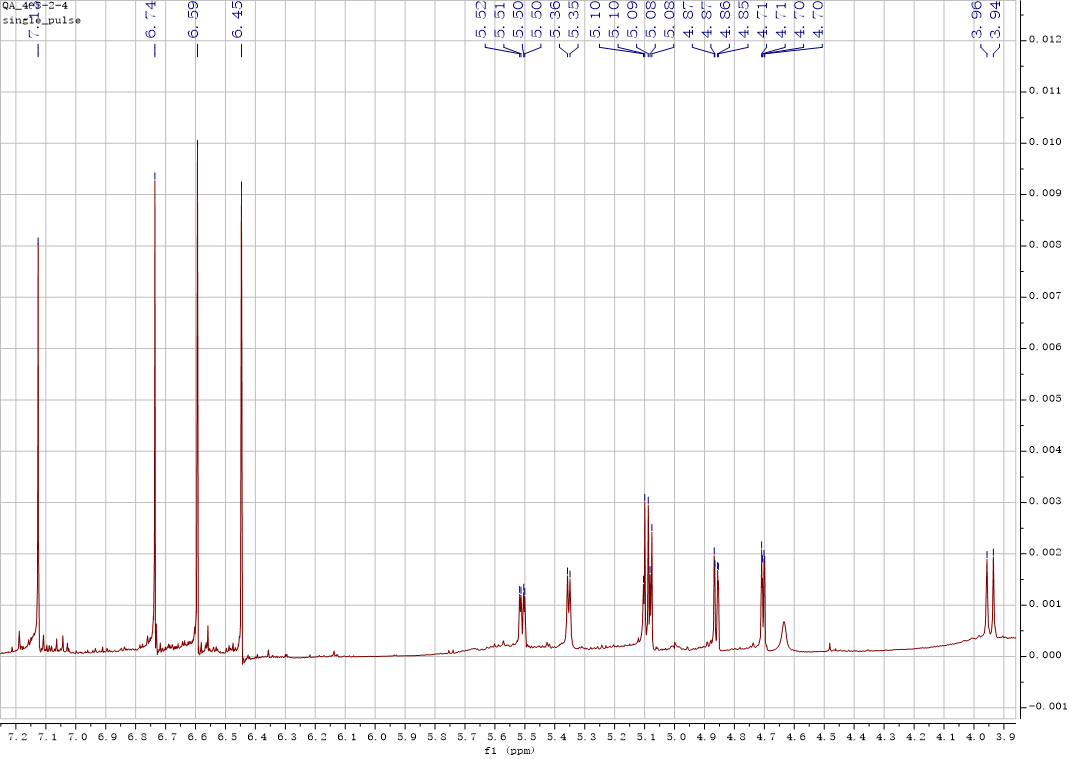

Supplement: Supplementary Materials — are provided files including the NMR spectra of six compounds isolated from QA. Figure S1-1: 1H-NMR spectrum of compound 1 (600 MHz, Acetone-d6+D2O); Figure S1-2: 13C-NMR spectrum of compound 1 (150 MHz, DMSO- d6+D2O); Figure S2-1: 1H-NMR spectrum of compound 2 (600 MHz, Acetone-d6); Figure S2-2: 13C-NMR spectrum of compound 2 (150 MHz, Acetone-d6); Figure S3-1: 1H-NMR spectrum of compound 3 (600 MHz, Acetone-d6); Figure S3-2: 13C-NMR spectrum of compound 3 (150 MHz, Acetone-d6); Figure S4-1: 1H-NMR spectrum of compound 4 (600 MHz, Acetone-d6); Figure S4-2: 13C-NMR spectrum of compound 4 (150 MHz, Acetone-d6); Figure S5-1: 1H-NMR spectrum of compound 5 (600 MHz, Acetone-d6); Figure S5-2: 13C-NMR spectrum of compound 5 (150 MHz, Acetone-d6); Figure S6-1: 1H-NMR spectrum of compound 6 (600 MHz, Acetone-d6); and Figure S6-2: 13C-NMR spectrum of compound 6 (150 MHz, Acetone-d6). [file 9078475.f1.zip › Figure S6-1.png]

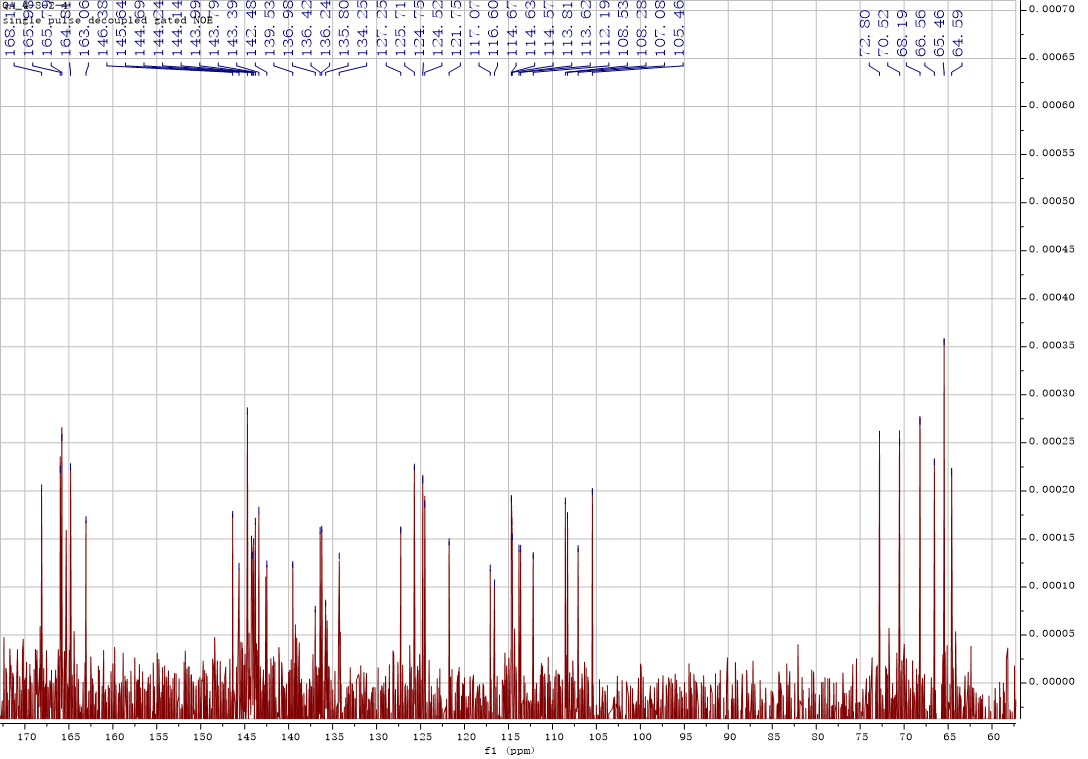

Supplement: Supplementary Materials — are provided files including the NMR spectra of six compounds isolated from QA. Figure S1-1: 1H-NMR spectrum of compound 1 (600 MHz, Acetone-d6+D2O); Figure S1-2: 13C-NMR spectrum of compound 1 (150 MHz, DMSO- d6+D2O); Figure S2-1: 1H-NMR spectrum of compound 2 (600 MHz, Acetone-d6); Figure S2-2: 13C-NMR spectrum of compound 2 (150 MHz, Acetone-d6); Figure S3-1: 1H-NMR spectrum of compound 3 (600 MHz, Acetone-d6); Figure S3-2: 13C-NMR spectrum of compound 3 (150 MHz, Acetone-d6); Figure S4-1: 1H-NMR spectrum of compound 4 (600 MHz, Acetone-d6); Figure S4-2: 13C-NMR spectrum of compound 4 (150 MHz, Acetone-d6); Figure S5-1: 1H-NMR spectrum of compound 5 (600 MHz, Acetone-d6); Figure S5-2: 13C-NMR spectrum of compound 5 (150 MHz, Acetone-d6); Figure S6-1: 1H-NMR spectrum of compound 6 (600 MHz, Acetone-d6); and Figure S6-2: 13C-NMR spectrum of compound 6 (150 MHz, Acetone-d6). [file 9078475.f1.zip › Figure S6-2.png]
